# Hermon-Taylor: *M. paratuberculosis* and Crohn’s Disease—The Book of Revelation According to John

**DOI:** 10.3390/pathogens10111469

**Published:** 2021-11-12

**Authors:** Coad Thomas Dow

**Affiliations:** McPherson Eye Research Institute, University of Wisconsin, 9431 WIMR, 1111 Highland Avenue, Madison, WI 53705, USA; ctomdow@gmail.com

Professor John Hermon-Taylor recently passed away on 16 October 2021. Prof. Hermon-Taylor was born in 1936, he received his B.A. degree in 1957 and his M.B. degree in 1960 from the Cambridge University. This London physician–scientist was an early elucidator of the zoonotic capacity of *Mycobacterium avium* ss. *paratuberculosis* (MAP). In 1998, John Hermon-Taylor (JHT) published an article in the British Medical Journal, a case report that featured a boy who, at seven years old, had cervical lymphadenitis. Five years later, when the same child presented with early Crohn’s disease, the archived lymphadenitis tissue was tested for MAP. It was positive, and the child was successfully treated with surgery followed by anti-mycobacterial drugs rifabutin and clarithromycin [1]. JHT’s work and advocacy continued to link MAP to Crohn’s disease [2] and he emphasized that, while humans are broadly exposed to MAP in dairy products, they are secondarily exposed to environmental MAP [3]. He predicted increasing MAP-related human disease with his “doomsday” article, a notably prescient forecast [4].

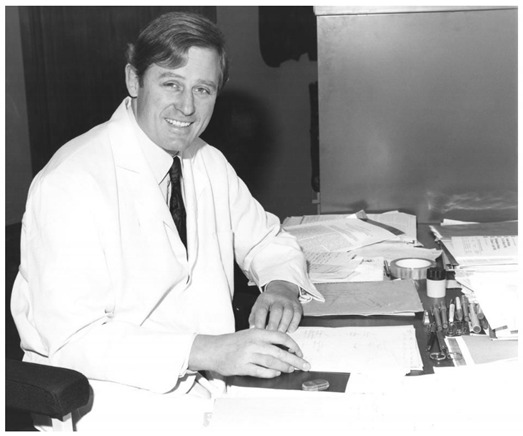



JHT’s easy manner and approachability encouraged many scientists to explore MAP’s contribution not only to Crohn’s disease, but to several other inflammatory and autoimmune diseases. Largely due to his foundational contributions, MAP scientists from around the world generated a 2017 consensus article that emphasized the zoonotic impact of MAP on human health [5].

Though the link of MAP zoonosis to Crohn’s disease has been a medical controversy for over one hundred years [6], validation of JHT’s effort has come from numerous recent studies showing Crohn’s disease resolution with anti-mycobacterial therapy targeted against MAP [7,8,9,10]. Moreover, MAP is now linked to an increasing list of inflammatory and autoimmune diseases [11]. To date, MAP has been causally associated with granulomatous diseases besides Crohn’s: sarcoidosis [12,13] and Blau syndrome [14]. Through molecular mimicry from mycobacterial protein elements, MAP is found to induce autoantibodies in autoimmune diabetes (T1D) [15], multiple sclerosis [16], autoimmune thyroiditis [17], lupus [18] and rheumatoid arthritis [19,20].

This writer predicts that, in the future, JHT’s contributions will be acknowledged throughout the world, perhaps securing him a commemorative place in Kensington Gardens beside the likes of Edward Jenner.
